# Leakage Via Aberrant Bile Duct Due to Cholangiocarcinoma

**DOI:** 10.1155/1998/61506

**Published:** 1998-12

**Authors:** R. Stanton, P. I. Craig, J. O. Jorgensen, D. L. Morris

**Affiliations:** 1 Department of Gastroenterology St. George Hospital Sydney Australia; 2 Department of Surgery St. George Hospital Sydney Australia; 3 Department of Surgery St. George Hospital Gray Street Kogarah New South Wales 2217 Australia

## Abstract

The case of a male who had an open cholecystectomy complicated by presistent bile leak from an aberrant bile duct is presented. The persistence and volume of bile leak resulted in subsequent investigation of the biliary tree which demonstrated a cholangiocarcinoma of the right hepatic duct. This case is presented as an unusual presentation of cholangiocarcinoma and to highlight the value of modern techniques in imaging the biliary tree.


*HPB Surgery*, 1998, Vol. 11, pp. 125-128
Reprints available directly from the publisher
Photocopying permitted by license only

© 1998 OPA (Overseas Publishers Association) N.V.
Published by license under
the Harwood Academic Publishers imprint,
part of The Gordon and Breach Publishing Group.
Printed in India.

*Case Report*

### Leakage *Via* Aberrant Bile Duct Due to Cholangiocarcinoma

R. STANTON [a,b], P. I. CRAIG [a], J. O. JORGENSEN [a,b] and D. L. MORRIS [b,*]

[a] *Department of Gastroenterology;* [b] *Department of Surgery, St. George Hospital, Sydney, Australia*

*(Received 30 April 1997)*

**The case of a male who had an open cholecystectomy complicated by presistent bile leak from an aberrant bile duct is presented. The persistence and volume of bile leak resulted in subsequent investigation of the biliary tree which demonstrated a cholangiocarcinoma of the right hepatic duct. This case is presented as an unusual presentation of cholangiocarcinoma and to highlight the value of modern techniques in imaging the biliary tree.**

*Keywords:* Aberrant bile duct, bile duct of Luschka, bile leak, cholangiocarcinoma, cholecysto-hepatic duct, choledochoscopy, endoscopic retrograde cholangio-pancreatography

### INTRODUCTION

In an 1863 paper, Luschka [1] first described the presence of aberrant bile ducts. They are characteristically less than 2 mm in diameter, drain a segment of the right lobe of the liver, and join the right hepatic, common hepatic or cystic duct [1-4]. These ducts rarely enter the gallbladder [5-8]. The incidence of the bile ducts of Luschka has been reported to be between 1% and 50% [2, 3, 5, 9, 10].

There is some confusion in the literature between the bile ducts of Luschka and cholecysto-hepatic ducts. The basic difference between these two types of aberrant bile duct appears to be that cholecysto-hepatic ducts, unlike the bile ducts of Luschka, drain into the gallbladder lumen.

This report presents a patient with an underlying cholangiocarcinoma who had an open cholecystectomy complicated by a persistant bile leak from an aberrant bile duct. The case is presented as the clinical course, investigation and treatment highlight many complex issues in modern hepato-biliary surgery.

### CASE REPORT

An 84 year old man had an open cholecystectomy for acute calculous cholecystitis. Intraoperative cholangiography was not performed.

The following day he developed increasing upper abdominal pain associated with jaundice

*Correspondence to: D. L. Morris, Department of Surgery, St. George Hospital, Gray Street, Kogarah, New South Wales, Australia, 2217.

and fever. He had a mild neutrophil leukocytosis, his liver function test (LFTs) were cholestatic, and his serum amylase was normal. The common bile duct (CBD) diameter was 6.8 mm on abdominal ultrasound, with no dilatation of the intrahepatic ducts seen. No calculi were seen.

On day 2 post-cholecystectomy, endoscopic retrograde cholangio-pancreatography (ERCP) showed a dilated biliary tree with both CBD and intrahepatic stones. Sphincterotomy delivered multiple small calculi. Occlusive ballon cholangiography demonstrated that the left intrahepatic system was normal, but demonstrated an irregular filling defect within a poorly filled right intrahepatic system. The aetiology of this was not apparent on imaging.

His sepsis and elevated LFTs improved over the course of the next 24 hours. However, his abdomen became noticeably distended and an abdominal CT scan showed a large amount of free intraperitoneal fluid with dilatation of segmental intrahepatic ducts on the right side of the liver. No intrahepatic calculi were seen.

A repeat ERCP on day 7 post-cholecystectomy again failed to demonstrate a normal right hepatic ductal system (see Fig. 1). A differential diagnosis of iatrogenic bile duct injury, haematobilia or cholangiocarcinoma was entertained. A percutaneous transhepatic cholangiogram (PTC) was perfomed and this demonstrated spillage of contrast from an aberrant bile duct into the gallbladder fossa; a large filling defect in the right hepatic duct was noted (see Fig. 2). An 8.5 F percutaneous self-retaining internal/external biliary drain was placed into the duodenum, with holes above and below the stricture. An intra-abdominal drain was also positioned into the fluid collection.

The patient's abdomen continued to distend despite biliary drainage of 200 ml per day. A progress abdominal CT on day 11 post-cholecystectomy showed a very large subhepatic collection. A second intra-abdominal drain was inserted under CT guidance and delivered 4.5 litres of bile.

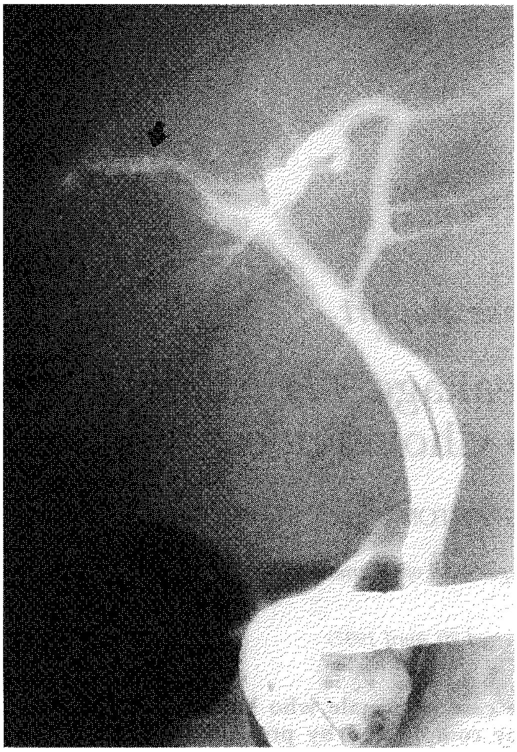

FIGURE 1 ERCP on day 7 post-cholecystectomy with catheter in right hepatic duct. Narrowed, irregular right hepatic duct seen (arrow).

On day 15 post-cholecystectomy, repeat cholangiogram via the trans-hepatic biliary drain confirmed a persisting filling defect in the right hepatic duct. Therefore, a 12 F percutaneous choledochoscope was introduced over a guidewire into the right duct system. A long malignant-appearing stricture involving the right hepatic duct was identified and biopsied. The left intrahepatic biliary tree and CBD were confirmed to be normal. Histopathology reported high grade dysplasia with suspicion of adenocarcinoma. Despite ongoing percutaneous stenting of the stricture, the intra-abdominal bile leak continued.

As all conservative measures to control his bile fistula had failed, excisional surgery as a means of palliation, and possible cure, was contemplated. At day 40 post-cholecystectomy, the patient underwent a right hemihepatectomy,

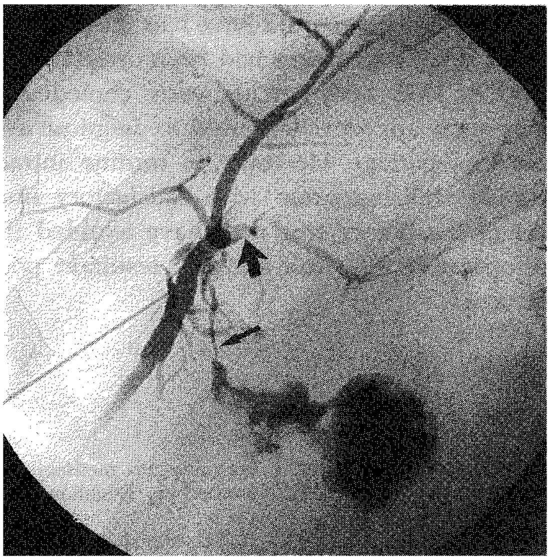

FIGURE 2 PTC on day 7 post-cholecystectomy showing proximal portion of filling defect in the right hepatic duct (thick arrows) and leakage from aberrant bile duct (thin arrows).

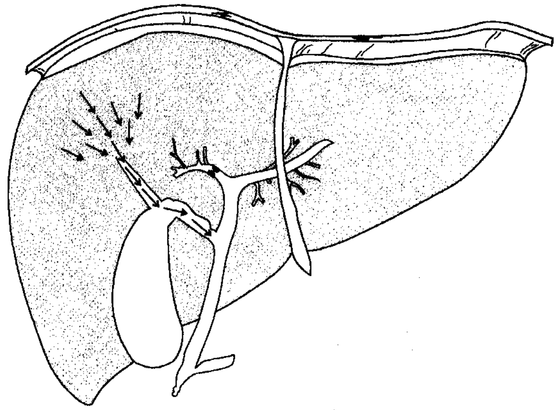

FIGURE 3a Pre-cholecystectomy showing malignant obstruction of the right hepatic duct and drainage of a segment of the right lobe of liver *via* the aberrant bile duct into the gallbladder or biliary tree.

with good macroscopic clearance of the tumour. Histopathology showed well differentiated, multifocal intrahepatic cholangiocarcinoma and early micronodular cirrhosis. He was discharged two weeks later.

He re-presented two weeks post discharge with jaundice and ascites. He eventually died of liver failure five weeks later.

### DISCUSSION

This report describes an unusual presentation for cholangiocarcinoma. Whilst small collections of bile are not uncommon after cholecystectomy, they usually resolve and are absorbed by the peritoneum [11, 12]. The right hepatic duct was obstructed by a malignant stricture. Therefore, before cholecystectomy, bile from a segment of the right lobe of liver drained *via* the aberrant bile duct into the biliary tree or gallbladder (see Fig. 3a). Cholecystectomy broke this internal bypass system resulting in persistent leakage from the aberrant bile duct into the peritoneal cavity (see Fig. 3b). The persistence and volume of the bile leak was one of the factors which precipitated further investigation of the biliary system and the subsequent discovery of the malignancy.

The question over whether the leak occurred from a damaged bile duct of Luschka or a cholecysto-hepatic duct had not been resolved. If the aberrant bile duct was a bile duct of Luschka, the bile leak could have potentially been avoided. Several authors have agreed that by dissecting as closely as possible to the gallbladder lumen during a cholecystectomy, the risk of damaging a bile duct of Luschka is decreased [2, 3]. These authors recommend ligation of a damaged leaking duct if recognised at the time of operation.

Usually one would expect percutaneous biliary drainage would allow a biliary leak to close. However, in this case, the leak persisted more than 30 days after placement of an external biliary drainage catheter and therefore operative intervention was required. Unfortunately, the resultant death of the patient from liver failure was due in part to his underlying cirrhosis.

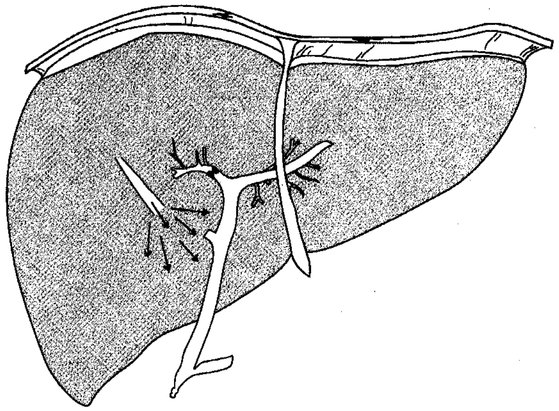

FIGURE 3b Post-cholecystectomy showing breaking of the internal bypass system resulting in leakage from the aberrant bile duct into the peritoneal cavity.

Percutaneous choledochoscopy is increasingly being used to diagnose and treat patients with complex benign and malignant biliary diseases [13, 14]. In this case, the diagnosis was confirmed by directly visualising and sampling the malignant stricture.

Finally, the value of an intra-operative cholangiogram in this case has to be addressed. Cholangiography would have identified stones in the CBD and may have noted the abnormal right hepatic duct system. This would have been extremely helpful as iatrogenic injury could have been excluded at the time of surgery. Also inspection and biopsy of the lesion may have been possible at the time of surgery by either flexible choledochoscopy, or by fine needle aspiration of any palpable mass. If a decision was made not to resect the cholangiocarcinoma, the method of cholecystectomy could have been modified. To avoid damaging gallbladder bed aberrant bile ducts, which in this case would have been under increased pressure, a partial cholecystectomy conserving the posterior wall could have been performed. Only a true cholecysto-hepatic duct would have had the potential to leak if such measures had been undertaken.

ERCP, PTC and percutaneous choledochoscopy were appropriately used to diagnose and treat this patient. However, a routine intraoperative cholangiogram with satisfactory imaging of the biliary tree may have avoided the postoperative bile leak and the resultant poor outcome.

### *References*

[1] Luschka, H. (1863). Die anatomie des menschlichen bauches. Tubingen: H. Lauppschen, Buchhandlung; 255.

[2] McQuillan, T., Manolas, S. G., Hayman, J. A. and Kune, G. A. (1989). Surgical significance of the bile ducts of Luschka. *Br. J. Surg.*, **76**, 696-698.

[3] Foster, J. H. and Wayson, E. E. (1962). Surgical significance of aberrant bile ducts. *Am. J. Surg.*, **104**, 14-19.

[4] Healey, J. E. and Schroy, P. C. (1953). Anatomy of the biliary ducts within the human liver. *Arch. Surg.*, **66**, 599-616.

[5] Hobsley, M. (1958). Intra-hepatic anatomy. *Br. J. Surg.*, **45**, 635-644.

[6] Braasch, J. W. (1958). Congenital anomalies of the gallbladder and bile ducts. *Clin. J. N. Am.*, **38**, 627.

[7] Mentzer, S. H. (1929). Anomalies of the bile ducts in man. *JAMA*, **93**, 1273-1279.

[8] Williams, C. and Williams, A. M. (1955). Abnormalities of the bile ducts. *Ann. Surg.*, **141**, 598.

[9] Goor, D. A. and Ebert, P. A. (1972). Anomalies of the biliary tree. *Arch. Surg.*, **104**, 302-309.

[10] Benson, E. A. and Page, R. E. (1976). A practical reappraisal of the anatomy of the extrahepatic bile ducts and arteries. *Br. J. Surg.*, **63**, 853-860.

[11] Gunn, A. A. (1969). Abdominal drainage. *Br. J. Surg.*, **56**, 274-276.

[12] Elboim, C. M., Goldman, L., Hann, L., Palestrant, A. M. and Silen, W. (1983). Significance of post-cholecystectomy subhepatic fluid collections. *Ann. Surg.*, **198**, 137-141.

[13] Ponchon, T., Genin, G., Mitchell, R., Henry, L., Bory, R. M., Bodnar, D. and Valette, P. J. (1996). Methods, indications and results of percutaneous choledochoscopy. A series of 161 procedures. *Ann. Surg.*, **223**(1), 26-36.

[14] Loke, R. H. T., Craig, P. I., Hatfield, A. R. W. and Lake, S. P. (1991). Experience with new percutaneous biliary endoscopes in the gallbladder and bile duct. *Gut*, **32**, 1226-1227.

